# Rheumatic Congestive Fever

**Published:** 1854-10

**Authors:** Edwin R. Maxson

**Affiliations:** Adams Centre, Jefferson Co., N. Y.


### ART. III.—*Rheumatic Congestive Fever.* By Edwin R. Maxson, M. D., Adams Centre, Jefferson Co., N. Y.

A rheumatic fever, of a bilious, congestive character, has been prevailing in this vicinity since November last; but mainly during the winter and spring months. During this time, I have treated about fifty cases.

As some of its complications have been rather peculiar, and learning, too, that sporadic cases, of a similar character, have occurred in various parts of the country, I here offer a general description of its character, symptoms, complications, &c., and also the treatment which I soon adopted, and with which I have had good reason to be well satisfied.

It may be proper here to state, that it is common for us to get bilious fevers, to some extent, in this locality, during spring and autumn.

The premonitory symptoms of this fever, when noticed, have been much, like those of our bilious fever. Loss of appetite, bitter taste of the mouth, coated tongue, and in some cases, catarrhal symptoms, with a slight cough and darting pains in various parts of the body.

These symptoms usually continue from four to six days; but have in many cases been so slight, as to pass almost unnoticed; till a chill, with violent pains in the head, back, and limbs, announces the attack. In those cases in which catarrhal symptoms, with a slight cough, had been a leading premonitory symptom, great difficulty of breathing usually began with the first chill, or congestive stage.

In those cases in which acute, darting pains in the limbs and various parts of the body was the leading premonitory symptom, severe pain, and inability to use the limbs, as in inflammatory rheumatism, have begun with the first reaction, or stage of febrile excitement.

In children under five years old, and in very old people, the first congestion has produced symptoms like hydrocephalus, and apoplexy; they remaining in a congestive state, nearly insensible, for several hours. And if there had been catarrhal symptoms, great difficulty of breathing; occasionally throwing from the lungs a bloody mucus, in a frothy state.

Usually, however, reaction would take place in from one to six hours, during which, high febrile symptoms usually prevailed: the next congestive stage coming on in twenty-four or forty-eight hours, attended with little or no coldness, but intolerable pain in the head, intolerance to light, great difficulty of breathing, and aggravation of the rheumatic symptoms: the limbs being swelled, the joints stiff, and in some cases entirely helpless; the head continuing hot, and the breathing difficult; only a slight respiratory murmur being detected by the ear. In more advanced stages, however, a crepitus, as in pneumonia, might be detected in one or both lungs.

In some cases, the brain would suffer most during the first chill, or congestion; the lungs during the second; and then finally locate in the limbs, as in acute inflammatory rheumatism.

Great gastric irritability existed in all the cases, and there was frequently obstinate vomiting.

Erysipelas of the face and scalp existed in a few of the cases.

Rheumatic inflammation of the brain, lungs, stomach, or limbs, existed in most of the cases; but congestion was the leading feature of all the cases.

The general cause of this epidemic, I am satisfied, was malarious, like that producing our bilious fevers. It commenced in November, on the lowest grounds, and moved slowly, but steadly, toward our highest range of land, which it had reached by April.

The cause of the rheumatic character of this fever I am disposed to attribute, mainly, to the open, damp, and exceedingly changeable weather, during the winter and spring months.

It was mostly over by June, but occasional cases have occurred during the summer months. I have now, Aug. 23d, one case of it, in a man over eighty.

*Treatment.* The principal indications in the treatment have appeared to be, *first*, to equalize the circulation; *secondly*, to allay gastric irritability; and *lastly*, to keep up reaction, and subdue rheumatic inflammation, when it existed, in the brain, lungs or limbs.

To equalize the circulation, and promote reaction, I have usually directed a warm foot-bath, and friction along the spine, with a strong infusion of capsicum in vinegar, having both continued, morning and evening, for three or four days, if the symptoms require it.

I have usually given a cathartic of calomel and oil; or pills hydg. mass, gr. vi, followed in five or six hours with castor oil; and applied a blister over the stomach before or immediately after the operation of the cathartic. The effects of the blister are immediate, lessening the pain in, and tendency to the brain and lungs; and generally lessening the rheumatic difficulty in the limbs.

To keep up reaction, lessen irritability, and overcome the congestive tendency, I have given the following:

℞ Quinine Sulph., gr. vi.
Pulv. Doveri, gr. iv.
" Jacobi, gr. iii. M.

Continued every four or six hours.

If the rheumatic inflammation of the brain, lungs, or limbs continues, after the congestive tendency is mainly arrested, I have usually given,

℞ Potass. Iodid., gr. x,

Well diluted, every six hours, alternating with the above powders.

With this treatment, I have generally been able to discharge my patients in eight or nine days: a few, however, have run on two or three weeks.

Two have terminated fatally. One, a child, four years old, terminated in hydrocephalus; and the other, an old lady, seventy or eighty, who got up from one attack, and was discharged; but sinking into a relapse, died in an apoplectic state, without any reaction having taken place.

ADAMS CENTRE, N. Y., Aug 24th, 1854.

